# Antibiotic susceptibility profiles of some *Vibrio *strains isolated from wastewater final effluents in a rural community of the Eastern Cape Province of South Africa

**DOI:** 10.1186/1471-2180-10-143

**Published:** 2010-05-14

**Authors:** Anthony I Okoh, Etinosa O Igbinosa

**Affiliations:** 1 Applied and Environmental Microbiology Research Group (AEMREG), Department of Biochemistry and Microbiology, University of Fort Hare, Private Bag X1314, Alice 5700, South Africa

## Abstract

**Background:**

To evaluate the antibiogram and antibiotic resistance genes of some *Vibrio *strains isolated from wastewater final effluents in a rural community of South Africa. *V. vulnificus *(18), *V. metschnikovii *(3), *V. fluvialis *(19) and *V. parahaemolyticus *(12) strains were isolated from final effluents of a wastewater treatment plant (WWTP) located in a rural community of South Africa. The disk diffusion method was used for the characterization of the antibiogram of the isolates. Polymerase chain reaction (PCR) was employed to evaluate the presence of established antibiotic resistance genes using specific primer sets.

**Results:**

The *Vibrio *strains showed the typical multidrug-resistance phenotype of an SXT element. They were resistant to sulfamethoxazole (Sul), trimethoprim (Tmp), cotrimoxazole (Cot), chloramphenicol (Chl), streptomycin (Str), ampicillin (Amp), tetracycline (Tet) nalidixic acid (Nal), and gentamicin (Gen). The antibiotic resistance genes detected includes *dfr18 *and *dfrA1 *for trimethoprim; *floR, tetA, strB, sul2 *for chloramphenicol, tetracycline, streptomycin and sulfamethoxazole respectively. Some of these genes were only recently described from clinical isolates, demonstrating genetic exchange between clinical and environmental *Vibrio *species.

**Conclusions:**

These results demonstrate that final effluents from wastewater treatment plants are potential reservoirs of various antibiotics resistance genes. Moreover, detection of resistance genes in *Vibrio *strains obtained from the wastewater final effluents suggests that these resistance determinants might be further disseminated in habitats downstream of the sewage plant, thus constituting a serious health risk to the communities reliant on the receiving waterbodies.

## Background

Antibiotic-resistant bacteria have been found in a surprisingly diverse range of environments, including human clinics, animal husbandry, orchards, aquaculture, food, sewage, chlorinated, and unchlorinated water supplies [1]. Antimicrobial resistance has become a major medical and public health problem as it has direct links with disease management [2]; and while antibiotics such as tetracycline, doxycycline, norfloxacin, ciprofloxacin and streptomycin may be used as an adjunct in rehydration therapy and are critical in the treatment of septicemia patient [3-5], resistance to many of these drugs in many pathogens including *Vibrio *pathogens such as *V. vulnificus, V. cholerae, V. fluvialis *and *V. parahaemolyticus *[6-8] have been documented.

Report of drug-resistant *V. cholerae *strains are appearing with increasing frequency [9]. Emergence of microbial resistance to multiple drugs is a serious clinical problem in the treatment and containment of the cholera-like diarrhoea, as reflected by the increase in the fatality rate from 1% to 5.3% after the emergence of drug-resistance strains in Guinea-Bissau during the cholera epidemic of 1996-1997 [10]. A genetic element, termed SXT element, which has properties similar to those of the conjugative transposons, was found to carry genes encoding resistance to sulfamethoxazole, trimethoprim and streptomycin in *V. cholerae *O139 and O1 strains isolated in India, but was not present in O1 strain obtained in 1994 from Rwandan refugees in Goma, Zaire [11]. Previous report showed that gene cassettes contained in class 1 integrons were distributed among different *V. cholerae *O-serotypes of mainly clinical origin in Thailand [12]. Also, the presence and transfer of SXT element and resistance gene in class 1 integrons have been studied in South Africa [13], which reported for the first time the presence of SXT element in *V. cholerae *O1 clinical isolates in Africa [13].

As the SXT genetic element plays a role in the acquisition of antibiotic resistance, it is important to also assess the presence of *sul2 *(encoding sulfamethoxazole resistance), *dfrA1 *(O1-specific trimethoprim resistance), *dfr18 *(O139 and non-O139 trimethoprim resistance) [14,15], and *strB *(streptomycin B resistance) gene in *V. cholerae *strains [16-18]. Waldor et al. [1996] identified in *V. cholerae *O1 and O139 an approximately 62 kb self-transmissible, chromosomally integrating genetic element, which was found to contain genes encoding resistance to sulphonamides, trimethoprim and streptomycin [11]. However, the antibiotic susceptibilities of organisms fluctuate spatially and temporally [19]. These susceptibilities have to be examined in order to better understand the organisms' epidemiological features [19].

To the best of our knowledge, no antibiotic resistance gene profile has been investigated in *Vibrio *species isolated from wastewater final effluents in the rural communities of South Africa, a country currently facing increasing pressure of water pollution from both domestic sewage and industrial wastewater, thus posing a threat to the public health of humans and ecological diversity of marine animals. As part of our ongoing surveillance study on aquatic microbial pathogens, we isolated some *Vibrio *pathogens [20], and in this paper, we report the antibiotic susceptibility patterns of the *Vibrio *isolates as well as the distribution of antibiotic resistance genes in the isolates.

## Results and Discussion

### Physicochemical analysis of final effluent quality

In our previous study [21] we reported some physicochemical parameters from the final effluents of a wastewater treatment facility (Table 1). Considerably high concentration of COD, nitrate, and orthophosphate were reported in the study [21]. The quality of the final effluent was consequently evaluated by other standards as reported in [21,22]. The final effluents qualities were not compliant to recommended standards for turbidity, COD, nitrate and orthophosphate (Table 1). This disqualifies the effluents for use in domestic activities and suggests that discharging such effluents into receiving watersheds could support eutrophication, with its attendant negative consequence [23].

**Table 1:** Seasonal and annual mean values of physicochemical qualities from the final effluent.

**Parameters**	**Final effluent**					
	
	**Range**	**Mean ± SD**	**Autumn**	**Summer**	**Winter**	**Spring**
	
pH	5.53 - 9.38	6.65 ± 0.97	6.40 ± 0.29^C^	7.03 ± 1.31^C^	6.10 ± 0.58^D^	6.70 ± 0.34^C^
Temperature (°C)	13.04 - 27.21	20.95 ± 4.37	19.82 ± 3.01^A^	24.73 ± 2.28^B^	15.24 ± 2.00^A^	20.98 ± 0.98^A^
Turbidity (NTU)	1.59 - 25.5	6.68 ± 5.73	6.25 ± 4.86^C^	9.64 ± 7.32^C^	3.81 ± 0.93^C^	3.68 ± 2.24^D^
TDS (mg/l)	121 - 244	144 ± 19.76	149.50 ± 0.54^A^	133.26 ± 6.80^A^	144.77 ± 10.68^B^	168.40 ± 42.48^B^
DO (mg/l)	1.16 - 9.46	5.02 ± 2	4.15 ± 0.90^C^	5.38 ± 2.73^A^	4.85 ± 1.25^C^	4.96 ± 1.56^B^
COD (mg/l)	10 - 975	126 ± 230.6	46.00 ± 41.69^A^	238.00 ± 333.71^A^	49.00 ± 26.92^A B^	34.82 ± 17.98^B^
NO_3_^- ^(mg/l)	4.4 - 18.8	10.43 ± 3.8	11.75 ± 8.14^A^	8.73 ± 2.08^A^	13.10 ± 0.95^A^	7.96 ± 5.22^A^
NO_2_^- ^(mg/l)	0.03 - 0.46	0.21 ± 0.12	0.12 ± 0.07^B^	0.19 ± 0.08^A B^	0.21 ± 0.15^B^	1.30 ± 1.85^A^
PO_4_^3- ^(mg/l)	0.12 - 4.3	2.02 ± 1.40	0.33 ± 0.18^A^	4.81 ± 0.58^A^	2.16 ± 1.71^A^	3.98 ± 0.13^A^

Values are means of triplicates ± Standard deviations (SD); Means with the same letter are not significantly different (*P *> 0.005). Summer (November to March); autumn (April to May); winter (June to August); spring (September to October)

TDS, Total dissolved solid; DO, Dissolved oxygen; COD, Chemical oxygen demand; NO_3_^-^, Nitrate; NO_2_^-^, Nitrite; PO_4_^3-^, Orthophosphate.

### Antibiogram profile

The susceptibilities of *V. vulnificus *(18 strains); *V. parahaemolyticus *(12 strains); *V. fluvialis *(19 strains) and *V. metschnikovii *(3 strains) to 21 different antibiotics by were examined. All the 52 isolates of *Vibrio *species were resistant to ampicillin and sulfamethoxazole, and sensitive to imipenem, meropenem and norfloxacin. *Vibrio fluvialis *showed 100%, 90%, 70% and 80% resistances to trimethoprim, penicillin, cotrimoxazole and streptomycin, respectively, and 92%, 82% 90% and 100% of cephalothin resistances were exhibited by *V. vulnificus, V. parahaemolyticus, V fluvialis *and *V*. *metschnikovii *respectively. The results reveal the high individual and multiple antibiotics resistance among the test *Vibrio *strains (Additional file 1). Previous studies have shown that streptomycin, rifampicin, kanamycin, tetracycline, polymyxin B were active against *Vibrio *species [24], but this was at variance with our findings where we observed resistances to streptomycin, tetracycline and polymyxin B in our *Vibrio *isolates. In this study, resistance to ampicillin was observed in all our *Vibrio *strains in difference to other studies that have been reported [25,26], but corroborated by the findings of French and coworker [27] who reported similar antibiotics susceptibility profile for *V. parahaemolyticus*.

An increase in multi-antibiotics resistance bacteria in recent years is worrisome and the presence of resistance genes in bacteria has further aided the transmission and spread of drug resistance among microbial pathogens [28]. Most studies on the antimicrobial susceptibility profiles of *Vibrio *species focus almost exclusively on clinical and/or food isolates with little information in the literature on those isolated from environmental sources such as treated municipal wastewater effluents. To our knowledge, this is the first study that specifically evaluated the antimicrobial susceptibility profile and detection of multiple antibiotics resistance genes of *Vibrio *strains isolated from treated municipal wastewater effluent in South Africa.

### The antibiotic resistance gene cluster and SXT element of *Vibrio *species strains

In an attempt to finding a relationship between the multidrug-resistance phenotypes of *V. vulnificus, V. metschnikovii, V. fluvialis *and *V. parahaemolyticus *and the presence of the SXT-like element, polymerase chain reaction experiments were carried out using specific primers for the presence of antibiotic resistance genes and the SXT element (Table 2). To ascertain the contribution of SXT to strain resistance profile, we analysed for the presence of *sul2, floR, dfr18, strB*, and *dfrA1*, typical clustered resistance genes, able to discriminate among SXT variant. Results revealed that some *V. vulnificus, V. metschnikovii, V. fluvialis *and *V. parahaemolyticus *contained one to six of the antibiotic resistance genes of SXT-like element (Additional file 1). The most abundant strain that harboured most of the antibiotic resistance genes and SXT element is *V. fluvialis*. Strains AL024, AL038, AL054 AL056 and AL009 lack SXT integrase, hence, the entire element. TMP, STR and COT resistance can then be associated with any other mobile element especially the class 1 integrons, already described in Africa, both in *V. cholerae *and *V. parahaemolyticus*. SXT-like element devoid of the resistance cluster could be represented by strain AL016, positive for the integrase but not for the gene cassettes.

**Table 2:** Sequence of primers used for detection of antibiotics resistance genes and the SXT element.

Primer	Sequence (5' to 3')	Target gene	Amplicon size (bp)	Reference
SXT-F	ATGGCGTTATCAGTTAGCTGGC	SXT integrase	1035	[16]
SXT-R	GCGAAGATCATGCATAGACC			
SUL2-F	AGGGGGCAGATGTGATCGC	*sul2*	625	[17]
SUL2-B	TGTGCGGATGAAGTCAGCTCC			
FLOR-F	TTATCTCCCTGTCGTTCCAGCG	*floR*	526	[35]
FLOR-2	CCTATGAGCACACGGGGAGC			
TMP-F	TGGGTAAGACACTCGTCATGGG	*dfr18*	389	[17]
TMP-B	ACTGCCGTTTTCGATAATGTGG			
TetA-F	GTA ATT CTG AGC ACT GTC GC	*TetA*	950	[36]
TetA-R	CTG CCT GGA CAA CAT TGC TT			
strB-F	GGCACCCATAAGCGTACGCC	*strB*	470	[12]
strB-R	TGCCGAGCACGGCGACTACC			
dfr1-F	CGAAGAATGGAGTTATCGGG	*dfrA1*	372	[35]
dfr1-B	TGCTGGGGATTTCAGGAAAG			

To date, there have been no reports on the antibiotic resistance genes in *V. vulnificus, V. metschnikovii, V. fluvialis *and *V. parahaemolyticus *isolated from wastewater final effluents in rural communities of South Africa. The PCR result showed the presence and prevalence of SXT-like elements (with an amplicon size of 1035 bp) in the *Vibrio *strains (Additional file 1). The SXT-like element encodes different types of antibiotic resistance genes, *floR *(526 bp), *sul2 *(625 bp), and *strB *(470 bp), which confer resistance to chloramphenicol (Chl), sulfamethoxazole (Sul), and streptomycin (Str), respectively (Additional file 1). Trimethoprim (Tmp) resistance genes were detected with the amplification of a 372 and 389 bp fragment of *dfrA1 *and *dfr18 *(Additional file 1). The molecular analysis of these genes has been previously carried out in *V. cholerae *O1 and O139 [18,29].

In this present study, all strains exhibited multiple resistances to five antibiotics. Ramachandran et al. [29] carried a study of 51 strains of *V. cholerae *for detection of antibiotic resistant genes and the SXT element belonging to the serogroups O1, O139, non O1 and non-O139, all strains were found to habour antibiotics resistant gene and showed resistances to ampicillin, furazolidone, nalidixic acid, streptomycin, Trimethoprim - sulfamethoxazole and Trimethoprim. Another study carried out in India between 1997 and 1998 involving a total number of 94 isolates of *V. cholerae *reported that 43 strains belonging to non-O1 and non-O139 serogroups contained plasmids that contributed to the multiple antibiotic resistances and exhibited resistances to ampicillin, neomycin, tetracycline, gentamicin, streptomycin, sulfonamide, furazolidone, and chloramphenicol [30]. Our findings corroborate the earlier work of Ramachandran et al. [29] who reported differences in the antibiotics resistance gene cluster in the SXT-like element in *V. cholerae *O1 and O139.

The *dfr18 *and *dfrA1 *genes cassettes coding for trimethoprim resistance, found among several of our isolates, have also been detected among the strains isolated in Thailand [10], and India [30]. Similarly, the *strB *gene for aminoglycoside resistance (streptomycin) found in our collection have been previously detected by Falbo et al. [17] in Albania and Italy in 1994, and Calcutta, India during the period 1997 to 1998 [30]. Previous uses of antibiotics in the earlier outbreaks may be partly responsible for the extensive increase in antibiotics resistances that we have observed in this study. It is unknown whether the isolates responsible for earlier and recent epidemics are of clonal origin. The association between the developments of resistance to trimethoprim, cotrimoxazole and streptomycin with large-scale use of antibiotics for the treatment and prophylaxis of cholera is well recognized [13,31]. Still, our demonstration of multiple-drug resistant non-cholera vibrios isolates showing resistance to all the antibiotics traditionally used to treat cholera is worrisome and could have a direct impact on the treatment of current and future cholera cases in South Africa and other countries to which this isolate may spread. Dalsgaard et al. [13] speculated that recent occasional unusually high mortality rate experienced during cholera outbreaks in some African countries could be associated with multiple-drug resistant O1 isolates carrying resistance gene located in SXT element.

Our findings thus showed that SXT element bearing drug resistance markers were fairly widely distributed in the *Vibrio *strains isolated from our study sites. It also revealed the frequency of occurrence of the gene cassettes, *floR, tetA, dfr18, strB, dfrA1*, and *sul2*. Given that there are increasingly reports of cholera-like diarrhoea being caused by non-vibrio cholera strains, it is important to monitor the distribution of SXTs in emerging *Vibrio *species.

## Conclusion

To the best of our knowledge, this is the first study that describes the detection of antibiotics resistance genes known to confer resistances to common classes of antibiotics in a rural community of South Africa. The mobile pool of resistance genes shared by bacteria of the wastewater effluents analyzed even included resistance genes that have only recently been described in clinical isolates, indicating genetic exchange between clinical and environmental bacteria. Further, detection of these newer resistance genes isolated from bacterial inhabitants of wastewater final effluents confirms that these determinants are released into the environment, which subsequently facilitates further dissemination among environmental bacteria. Moreover, it appeared that the wastewater purification processes operating in the wastewater treatment facility under study are not efficient enough to significantly reduce the spectrum of resistance genes that are detectable in the final effluents. PCR can be used effectively to detect antibiotics resistance genes and could be used for the surveillance of the spread of antibiotics resistance in epidemiological and environmental studies.

## Methods

### Study site

The Wastewater treatment facility is situated at geographical coordinates of 32°50'36''S, 26°55'00''E and approximately 1 km East of Alice town in the Eastern Cape Province of South Africa. The plant which has a design capacity of 2000 m^3^/day receives domestic sewage, some light industrial wastewater as well as run-off water, and treatment is based on the activated sludge system. The final effluent is discharged into the nearby Tyume River.

### Isolation and biochemical identification of *Vibrio *species

Sample collection methods and treatments of collected samples has been described in our previous work [20]. Aliquots of the plankton free and plankton associated samples were inoculated into alkaline peptone water (APW, Pronadisa) and incubated aerobically at 37°C for 18-24 h. Turbid cultures were streaked onto thiosulphate citrate bile salts sucrose (TCBS, Pronadisa) agar and incubated at 37°C for 24 h. Five to ten isolated colonies per plate were randomly picked from each sample and subsequently subcultured on fresh TCBS agar plates. The pure isolates were then subjected to Gram staining and oxidase test, and only Gram-negative, oxidase-positive isolates were selected for biochemical identification using API 20 NE kit. The strips were then read and the final identification was made using API lab plus software (bioMerieux, Marcy l'Etoile, France). Polymerase chain reaction (PCR) was used to confirm the identities of the *Vibrio *species using the species-specific primers described in our previous study [20].

### Bacterial strains

A total of 52 strains of *Vibrio *species were included in this study. Of these, 12 were *V. parahaemolyticus*, 18 were *V. vulnificus*, 19 were *V. fluvialis *and 3 were *V. metschnikovii*. These *Vibrio *species were isolated in our previous study from the final effluent of a rural wastewater treatment plant in the Eastern Cape Province of South Africa [20]. *V. parahaemolyticus *strain SABS PM ATCC Vbr 1, *V. vulnificus *DSM 10143, *V. fluvialis *DSM 19283 were used as the PCR positive control for *sul2*, *dfrA1, strB, floR, dfr18, tetA*, and SXT integrase. All strains were maintained in tryptic soy broth supplemented 30% glycerol and stored at -80°C.

### Antibiotic susceptibility test

Bacterial susceptibilities to the test antibiotics were performed by disk diffusion method using guidelines established by Bauer et al. [32] and recommended by Clinical and Laboratory Standards Institute [33] using commercial antibiotics discs. A total of 21 antibiotic discs (Mast Diagnostics, Merseyside, United Kingdom) which includes ampicillin (25 μg), cotrimoxazole (25 μg), amikacin (30 μg), imipenem (10 μg), erythromycin (15 μg), meropenem (10 μg), streptomycin (25 μg), chloramphenicol (30 μg), ciprofloxacin (5 μg), cephalothin (30 μg), nalidixic acid (30 μg), tetracycline (30 μg), trimethoprim (30 μg), norfloxacin (10 μg), sulfamethoxazole (25 μg), gentamicin (10 μg), neomycin (30 μg), penicillin G (10 unit), nitrofurantoin (200 μg), polymyxin B (300 units) and cefuroxime (30 μg) were employed. Characterization of the resistance or susceptibility profile of the isolates was determined by measuring inhibitory zone and then compared with the interpretative chart to determine the sensitivity of the isolates to the antibiotics.

### Isolation of genomic DNA

Genomic DNA was extracted following a modified scheme of Maugeri et al. [34] Single colonies of *Vibrio *species strains grown overnight at 37°C on TCBS agar plates were picked, suspended in 200 μl of sterile Milli-Q PCR grade water (Merck, SA) and the cells were lysed using Dri-block DB.2A (Techne, SA) for 15 min at 100°C. The cell debris was removed by centrifugation at 11, 000 × *g *for 2 min using a MiniSpin micro centrifuge (Merck, SA). The cell lysates (10 μl) were used as template in the PCR assays immediately after extraction placed on ice for 5 min or following storage at -80°C. Sterile Milli-Q PCR grade water (Merck, SA) was included in each PCR assay as negative control.

### PCR amplification assay

Polymerase chain reaction (PCR) was used to detect antibiotic resistant genes in the *Vibrio *species using the specific primer pairs and PCR conditions for detection of the SXT integrase, *floR, strB, sul2, dfrA18, tetA and dfrA1 *are listed in Table 2. All reactions were set in 50 μl volume of reaction buffer containing 0.05 unit/μl *Taq *polymerase as directed by the manufacturer (Fermentas Life Sciences). Cycling conditions (Bio-Rad My Cycler™ Thermal Cycler) were as follows; initial denaturation at 94°C for 2 min was followed by 35 cycles of 94°C for 1 min, 60.5°C for 1 min and 72°C for 1 min with a final extension at 72°C for 10 min and cooling to 4°C. Electrophoresis of amplicons was performed with 1% agarose gel (Hispanagar, Spain) containing Ethidium Bromide (EtBr) (Merck, SA) with 0.5 mg/L for 1 h at 100 V in 0.5× TAE buffer (40 mM Tris-HCl, 20 mM Na-acetate, 1 mM EDTA, pH 8.5) and visualized under an UV transilluminator (BioDoc-It System, UVP Upland, CA 91786, USA).

## Authors' contributions

AIO: conceived of the study, participated in its design, provided technical support and helped to prepare the manuscript. EOI: participated in the study design, carried out the experimental work, and drafted the manuscript. All authors read and approved the final version of the manuscript.

## Supplementary Material

Additional file 1 **Phenotypic and genotypic characterization of *Vibrio *strains and their antibiotics resistance genes**. Supplemental table. Click here for file
