# Supplementary material for: Antibiotic susceptibility profiles of some Vibrio strains isolated from wastewater final effluents in a rural community of the Eastern Cape Province of South Africa
Source: BMC Microbiol. 2010 May 14;10:143. doi: 10.1186/1471-2180-10-143 (PMC2877687; doi:10.1186/1471-2180-10-143)
Supplement: Additional file 1 — Phenotypic and genotypic characterization of Vibrio strains and their antibiotics resistance genes. Supplemental table. [file 1471-2180-10-143-S1.DOC]

Table 3. Phenotypic and genotypic characterization of *Vibrio* strains and their antibiotics resistance genes.

| **Isolate Code** | **Name of species** | **Antibiotic resistance pattern** | **Strain(s) showing presence of gene encoding** | | | | | | |
| --- | --- | --- | --- | --- | --- | --- | --- | --- | --- |
|  |  |  | *floR* | *dfr18* | *tetA* | *strB* | *dfrA1* | *sul2* | SXT integrase |
| AL028 | *Vibrio vulnificus* | 1 | + | - | *-* | *-* | *-* | *-* | + |
| AL 010 | *Vibrio vulnificus* | 3 | + | - | + | - | - | - | - |
| AL016 | *Vibrio vulnificus* | 1 | - | - | - | - | - | - | + |
| AL024 | *Vibrio vulnificus* | 2 | - | - | + | - | - | - | - |
| AL026 | *Vibrio vulnificus* | 4 | + | + | - | + | - | - | + |
| AL001 | *Vibrio vulnificus* | 3 | + | - | - | + | - | - | + |
| ALO15 | *Vibrio vulnificus* | 2 | + | + | + | + | - | + | + |
| AL029 | *Vibrio vulnificus* | 8 | - | + | - | + | - | + | + |
| AL038 | *Vibrio vulnificus* | 6 | - | + | - | + | - | - | - |
| AL018 | *Vibrio vulnificus* | 3 | - | + | - | - | - | - | + |
| AL039 | *Vibrio vulnificus* | 7 | + | - | - | - | + | + | + |
| AL041 | *Vibrio vulnificus* | 4 | + | + | + | + | - | + | + |
| AL044 | *Vibrio vulnificus* | 1 | + | + | - | - | - | - | + |
| AL054 | *Vibrio vulnificus* | 2 | + | - | + | + | + | - | - |
| AL042 | *Vibrio vulnificus* | 2 | + | - | + | + | + | - | + |
| AL048 | *Vibrio vulnificus* | 4 | - | - | - | + | - | + | + |
| AL011 | *Vibrio vulnificus* | 1 | - | + | - | - | - | - | + |
| AL056 | *Vibrio vulnificus* | 2 | - | - | + | + | - | - | - |
| AL012 | *Vibrio metschnikovii* | 5 | - | - | - | - | - | + | + |
| AL016 | *Vibrio metschnikovii* | 5 | + | + | - | + | - | + | + |
| AL023 | *Vibrio metschnikovii* | 1 | + | - | + | + | + | + | + |
| AL014 | *Vibrio fluvialis* | 1 | + | + | - | + | - | + | + |
| AL002 | *Vibrio fluvialis* | 7 | - | + | - | + | + | + | + |
| AL025 | *Vibrio fluvialis* | 6 | + | + | - | + | + | + | + |
| AL037 | *Vibrio fluvialis* | 7 | - | + | - | + | + | + | + |
| AL033 | *Vibrio fluvialis* | 5 | + | + | - | - | + | + | + |
| AL013 | *Vibrio fluvialis* | 1 | + | + | - | + | + | + | + |
| AL024 | *Vibrio fluvialis* | 6 | + | + | - | - | + | + | + |
| AL027 | *Vibrio fluvialis* | 6 | - | + | - | + | + | + | + |
| AL029 | *Vibrio fluvialis* | 2 | + | + | + | + | + | + | + |
| AL034 | *Vibrio fluvialis* | 3 | + | + | - | + | + | + | + |
| AL036 | *Vibrio fluvialis* | 1 | + | + | - | + | + | + | + |
| AL040 | *Vibrio fluvialis* | 1 | + | + | - | + | + | + | + |
| AL051 | *Vibrio fluvialis* | 1 | - | + | - | + | + | + | + |
| AL053 | *Vibrio fluvialis* | 1 | + | + | + | + | + | + | + |
| AL019 | *Vibrio fluvialis* | 1 | + | + | - | + | + | + | + |
| AL004 | *Vibrio fluvialis* | 5 | - | + | - | + | + | + | + |
| AL006 | *Vibrio fluvialis* | 2 | - | + | + | - | + | - | + |
| AL022 | *Vibrio fluvialis* | 8 | + | + | - | + | + | + | + |
| AL031 | *Vibrio fluvialis* | 7 | + | + | - | - | + | - | + |
| AL003 | *Vibrio parahaemolyticus* | 5 | - | - | - | + | + | - | + |
| AL008 | *Vibrio parahaemolyticus* | 4 | - | + | - | - | - | + | + |
| AL014 | *Vibrio parahaemolyticus* | 4 | - | + | - | + | - | + | + |
| AL017 | *Vibrio parahaemolyticus* | 6 | + | + | - | - | - | - | + |
| AL028 | *Vibrio parahaemolyticus* | 1 | + | - | - | + | + | + | + |
| AL009 | *Vibrio parahaemolyticus* | 6 | - | + | - | + | - | - | - |
| AL030 | *Vibrio parahaemolyticus* | 7 | + | + | - | + | - | + | + |
| AL032 | *Vibrio parahaemolyticus* | 7 | + | - | - | + | + | + | + |
| AL043 | *Vibrio parahaemolyticus* | 7 | - | + | + | + | - | + | + |
| AL045 | *Vibrio parahaemolyticus* | 7 | + | - | - | + | + | + | + |
| AL049 | *Vibrio parahaemolyticus* | 6 | - | + | - | - | - | + | + |
| AL055 | *Vibrio parahaemolyticus* | 2 | - | - | + | + | - | - | + |
| Positive controls | *V. vulnificus* DSM 10143 | 9 | + | + | - | + | + | + | + |
|  | *V. fluvialis* DSM 19283 | 10 | + | + | - | + | + | + | + |
|  | *V. parahaemolyticus* strain SABS PM ATCC Vbr 1 | 11 | + | + | - | + | + | + | + |

Legend: 1- COT, CHL, NAL, ERY, CXM, AK, NAL, PEN

2- TET, CIP, ERY, PB, CXM, TMP

3- CIP, AK, ERY, NAL, GEN

4- ERY, CHL, NIT, GEN, TMP

5- AK, TMP, ERY, CHL, STR, NEO

6- PB, GEN, STR, PEN, CHL, NAL

7- GEN, PEN, COT, TMP, CHL, ERY

8- TMP, ERY, PEN, NAL, STR, CHL

9- NAL, STR, TMP, COT, PB, NEO

10- CIP, GEN, STR, TMP, NEO, NAL, NOR, CHL, COT, PB

11- TMP, STR, COT, NEO, PB

CHL, chloramphenicol; CIP, ciprofloxacin; GEN, gentamicin; NAL, nalidixic acid; NEO, neomycin; NOR, norfloxacin; STR, streptomycin; COT, cotrimoxazole; TET, tetracycline; TMP, trimethoprim; ERY, erythromycin; CXM, cefuroxime; AK, amikacin; PB, polymyxin B; PEN, penicillin G.
